# Single senescent cell sequencing reveals heterogeneity in senescent cells induced by telomere erosion

**DOI:** 10.1007/s13238-018-0591-y

**Published:** 2018-11-12

**Authors:** Huanyin Tang, Anke Geng, Tengjiao Zhang, Chen Wang, Ying Jiang, Zhiyong Mao

**Affiliations:** 1 Clinical and Translational Research Center of Shanghai First Maternity & Infant Hospital, Shanghai Key Laboratory of Signaling and Disease Research, School of Life Sciences and Technology, Tongji University, Shanghai, 200092 China; 2 School of Life Sciences and Technology, Tongji University, Shanghai, 200092 China

**Dear Editor,**

Over a half-century ago, Dr. Leonard Hayflick described the phenotype of a finite lifespan for human fibroblasts being passaged in *in vitro* cell culture (Hayflick et al., 1961), a phenomenon today known as replicative cellular senescence. Cellular senescence has been defined as a state in which cells lose their potential to divide and are permanently arrested in either the G_1_, or arguably the G_2_ stage of the cell cycle (Mao et al., 2012). In addition to replicative cellular senescence—which is induced by large amounts of DNA damage at telomeres due to loss of the specialized T-loop structure—exogenous sublethal stresses such as ionizing radiation, genotoxic chemicals or hyper-activated oncogenes may also trigger a similar form of senescence, stress induced premature cellular senescence (SIPS). Cells in the states of replicative senescence and SIPS share a number of common features such as changes in morphology, secretion of inflammatory factors and an increase in β-galactosidase activity. The induction of cellular senescence is mediated by either Rb or p53, two critical tumor suppressors, and represents a potent anti-tumor mechanism (Campisi, 2013). Cellular senescence is also beneficial to injured tissues or organs by secreting inflammatory factors, termed the senescence associated secretory phenotype (SASP), to promote tissue repair and the clearance of senescent cells. Paradoxically, if senescent cells are not promptly removed by NK cells and macrophages (Krizhanovsky et al., 2008), the persistent secretion of SASP factors poses a great threat to tissue homeostasis and may promote tumorigenesis, angiogenesis and even metastasis (Rao et al., 2016). Additionally, the paracrine SASP signaling may also drive the neighboring, young, healthy cells into cellular senescence through the TGF- beta and IL-1 signaling (Acosta et al., 2013). Therefore, although senescent cells are relatively rare *in vivo*, they may cause tremendous damage to the surrounding tissues or organs. Indeed, recently developed methods of clearing senescent cells *in vivo* using mouse models have greatly improved the healthy lifespan of rodents, suggesting that targeting senescent cells holds the potential to delay the onset of various age-related pathologies (Xu et al., 2018). The clinical applications of senescent cell-targeting agents, termed senolytics, will be gated by the specificity, efficacy and toxicity of these compounds. Ideally, such chemicals should selectively ablate all types of senescent cells with minimal or no damage to young normal cells. However, although very promising, no senolytic compounds developed to date are able to eliminate all types of senescent cells; further they retain a low level of toxicity to young normal cells (Lamming et al., 2013). Therefore, understanding whether senescent cells induced by either exogenous or endogenous genotoxic stresses, or senescent cells induced by the same stress are identical or heterogeneous would help better achieve the goal of selectively eliminating senescent cells. Senescent cells are generally enlarged in volume, and flattened in morphology if they are fibroblast. They also retain a high level of DNA damage in the nuclei (Muñoz-Espín et al., 2014). At molecular level, p16^INK4a^ and SASP are often employed as markers to identify senescent cells. However, most previous research was conducted at the population level. Whether senescent cells vary between single cells remains to be further determined. Intriguingly, a recent report analyzing the expression of SASP at single cell level reveals that different senescent cells have distinct patterns of SASP expression (Wiley et al., 2017). The report indicates that the observed high level of SASP might result from a small population of senescent cells secreting large amounts of SASP, indicating that senescent cells might be a mixture of single cells with different expression signatures. Nevertheless, no study has yet been performed to systematically examine the heterogeneity of replicatively cellular senescent cells or SIPS cells at a single cell level. Here we employed droplet-based single-cell mRNA sequencing (Drop-seq) (Macosko et al., 2015), which is a powerful tool for analyzing the mRNA expression level of a large number of single cells, to compare the expression signatures at single cell level.

To examine the change of heterogeneity during replicative cellular senescence, we cultured HCA2 fibroblasts to replicative senescence. Senescence was confirmed by increased number of β-gal positive cells and significantly reduced rate of EdU incorporation (Fig. S1A). Then we measured 1,200 single cell transcriptomes of young quiescent cells at a population doubling (PD) number of 38, middle age quiescent cells (PD = 48) and replicatively senescent (PD = 71) cells by Drop-seq (Fig. 1A). After filtering cells with low read count and batch effect correction, principal component analysis (PCA) was performed on normalized highly variable genes. Then we used a graph-based clustering approach to cluster the cells and visualize the cells by *t*-distributed stochastic neighbor embedding (*t*-SNE) based on statistically significant principal components.

**Figure 1:**
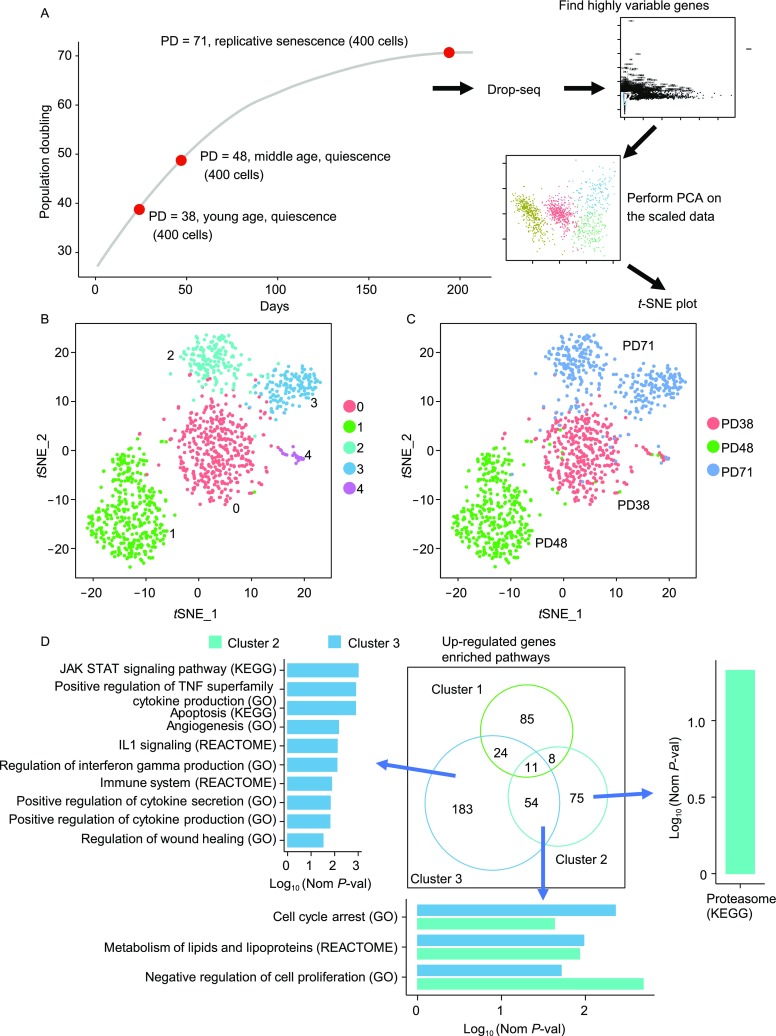
**Change of heterogeneity and transcriptome profile of HCA2 fibroblasts with replicative senescence**. (A) Schematic of replicatively senescent HCA2 fibroblast single-cell transcriptomics. (B) *t*-distributed stochastic neighbour embedding (*t-*SNE) plot of Drop-seq mRNA sequencing data from PD38 quiescent cells, PD48 quiescent cells and replicatively senescent cells. Cells were clustered into 5 distinct sub-populations. (C) Same as in (B), with cells color-coded by sample of origin. (D) Enriched pathways of up-regulated genes of cluster 1–3, the length and color of the bar show the significance of each cluster’s enriched pathways. Left barplot. Cluster 3-specific enriched pathways of cluster 3’s up-regulated genes. Bottom barplot. Cluster 3 and cluster 2 common enriched pathways of cluster 2’s and cluster 3’s up-regulated genes. Right barplot. Cluster 2-specific enriched pathways of cluster 2’s up-regulated genes

Our analysis splits the cells into 5 distinct sub-populations (Fig. 1B). Representative up-regulated marker genes of each cluster show that cluster 1, which mainly composed of quiescent cells, enriched ribosome associated genes, cluster 2 and cluster 3 exhibited upregulation of SASP genes, angiogenesis associated genes and apoptosis associated genes (Fig. S2A). Cluster 4 was composed of proliferating cells with a transcriptome profile enriched for the expression of cell cycle associated genes (e.g., MKI67, TOP2A and CENPE, Fig. S2B), so we excluded this cluster at downstream analysis. We compared this *t*-SNE plot to the *t*-SNE plot with cells color-coded to reflect sample of origin (Fig. 1C). Although cluster 0 and cluster 1 are all quiescent cells, they still could be split into two distinct clusters with low heterogeneity in each cluster. Interestingly, replicatively senescent cells were partitioned into 2 distinct sub-populations (Fig. 1C). So in replicatively senescent cells, heterogeneity does exist between cells.

In order to identify transcriptome level changes during replicative cellular senescence we used cluster 0 as control and examined differentially expressed genes between clusters. We applied a Wilcoxon rank sum test to find differentially expressed genes and a Bonferroni correction procedure to adjust for multiple comparisons. Genes were qualified as significantly differentially expressed at the adjusted *P* value < 0.05 and log_2_ fold change of >0.25 up- or down-regulated threshold. In replicatively senescent cells, cluster 3 had approximately two times more up-regulated genes and less down-regulated genes (Fig. S3A). In addition, there were also many statistically differentially expressed genes shared across all clusters, even between quiescent and senescent cells, and more than 70% genes of up- or down-regulated genes of cluster 2 were shared with cluster 3 but cluster 3 had more cluster 3-specific changed genes than those in cluster 2 (Fig. S3B). These cluster 3 specific genes may contribute to the heterogeneity of replicatively senescent cells.

To further investigate the transcriptome difference between clusters, we employed gene set enrichment analysis (GSEA) on up- and down-regulated genes of each cluster to identify enriched pathways in gene ontology biological process, Kyoto encyclopedia of genes and genomes (KEGG) and reactome pathway database (REACTOME) gene sets. As with the difference in differentially expressed genes number, cluster 3 also had more up-regulated gene enriched pathways and less down-regulated gene enriched pathways compared to those in cluster 2 (Fig. S3C). And there were also some enriched pathways shared across cluster 2 and cluster 3. They included up-regulated genes of cell cycle arrest, lipid metabolism and down-regulated genes of ribosome biogenesis, translation and metabolism of protein. In addition to common enriched pathways, cluster 3 had more senescence associated enriched pathways compared to cluster 2 in up-regulated genes and fewer senescence associated enriched pathways in down-regulated genes. Many cluster 3 up-regulated genes were enriched in pathways such as angiogenesis, immune system, IL1 signaling, SASP production and secretion, apoptosis and wound healing. We only observed the senescence associated proteasome pathway enriched in up-regulated genes of cluster 2 (Figs. 1D and S3D). But in down-regulated genes, cluster 1 had more enriched functions including lysine degradation, base excision repair, RNA methylation and telomere maintenance via telomerase. Cluster 2 had more enriched functions including regulation of autophagy, immune response, apoptosis, chromosome organization and DNA repair in down-regulated genes (Fig. S3D). Based on the GSEA analysis results, we propose that a fraction of replicatively senescent cells played distinct roles from others. These cells produce more SASP factors, particularly those of TGF super family, and activate IL-1 signaling, thus inducing more SASP which may prompt adjacent cells into senescence by paracrine SASP signaling. Higher secretion of SASP factors is also associated with an enhanced immune response, leading to inflammation which may promote tumorigenesis (Acosta et al., 2013).

We then set out to reveal the potential change in heterogeneity and transcriptome in stress-induced premature senescent (SIPS) cells. So we used Drop-seq to sequence the transcriptomes of 400 SIPS cells. Quiescent HCA2 cells at PD38 were irradiated at a dosage of 50 Gy. Twenty days later, the senescent phenotype was confirmed by β-gal staining and the EdU incorporation assay (Fig. S1B). After the analysis of single cell transcriptome of SIPS cells, we observed that the SIPS cells had low heterogeneity between cells, compared to replicatively senescent cells (Fig. 2A and 2B). Most SIPS cells were clustered into cluster 1.

**Figure 2:**
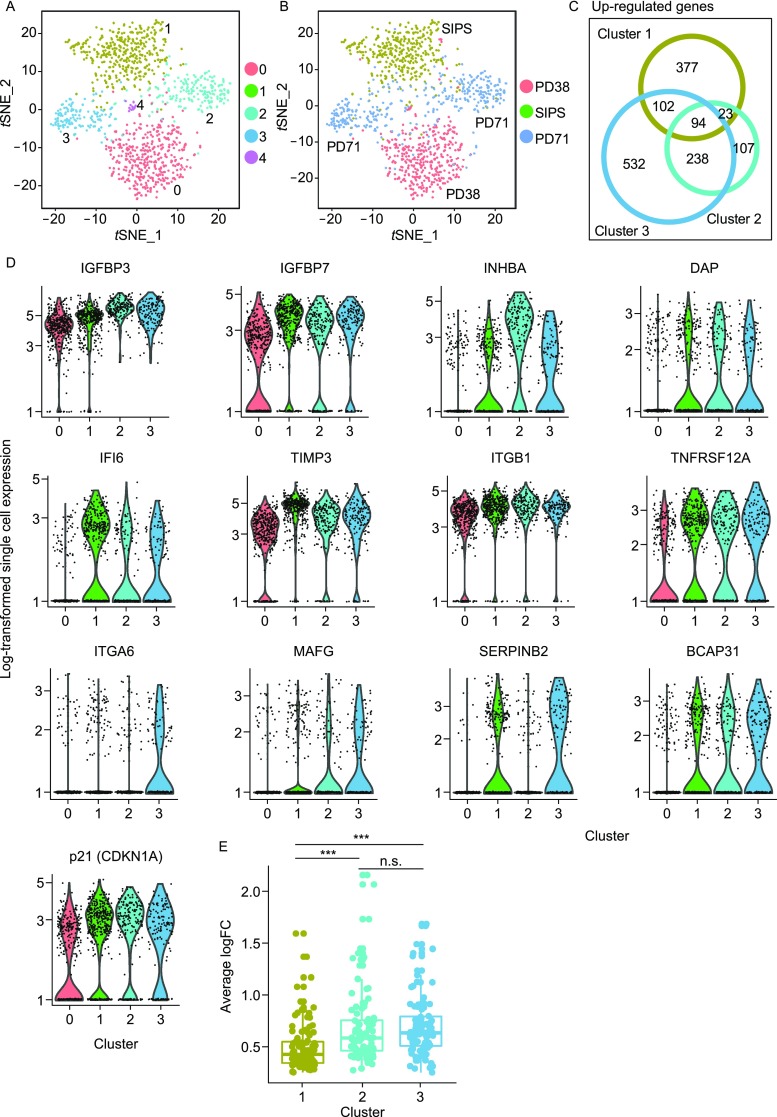
**Change of heterogeneity of HCA2 fibroblasts with SIPS**. (A) *t*-distributed stochastic neighbour embedding (*t*-SNE) plot of Drop-seq mRNA sequencing data from PD38 quiescent cells, replicatively senescent cells and 50 Gy X-ray induced senescent cells from the PD38 quiescent population. Cells were clustered into 5 distinct sub-populations. (B) Same as in (A), with cells color-coded by sample of origin. (C) Venn diagram of up-regulated genes among cluster 1–3. (D) Representative senescence core signature genes expression of each cluster. (E) Average fold changes of senescence core signature genes

It has been proposed that eliminating senescent cells may alleviate senescence induced physical dysfunction and increase lifespan (Xu et al., 2018). Thus, in order to identify the core genes which define the senescence signature and may serve as markers for the clearance of senescent cells, we also used PD38 quiescent cells (cluster 0) as control and performed differential expression analysis of each cluster. The senescence core signature was defined by up-regulated genes shared across 2 clusters of replicatively senescent cells and SIPS cells. The senescence core signature contained 94 up-regulated genes (Fig. 2C). These genes included classical senescence marker (p21), members of SASP (IGFBP3, IGFBP7), genes involved in apoptotic process (INHBA, DAP, etc.), response to cytokine (IFI6, TIMP3, etc.), angiogenesis (ITGB1, TNFRSF12A), response to wounding (ITGA6, MAFG, etc.) and regulation of proteolysis (SERPINB2, BCAP31, etc.) (Fig. 2D). Cluster 2’s and cluster 3’s average fold changes of senescence core signature genes are significantly higher than those in cluster 1. But between cluster 2 and cluster 3, there was no significant difference in average fold changes of senescence core signature genes (Fig. 2E). We also examined the transcriptome difference between telomere erosion and ionizing radiation induced senescence. By comparing the up-/down-regulated genes enriched pathways of cells in the state of RS and SIPS, we observed fewer common up-regulated genes. The up-regulated genes in RS cells were enriched in cytokine production, angiogenesis and IL1 signaling, while in SIPS cells up-regulated genes were enriched in VEGF and the NOTCH1 signaling, which is critical to paracrine secretion in senescent cells (Hoare et al., 2016). SIPS cells were associated with negative regulation of molecular function and catalytic activity (Fig. S4A). Although RS cells and SIPS cells shared several common down-regulated pathways including ribosome biogenesis, translation and metabolism of protein, RS cells have more down-regulated gene enriched pathways than SIPS cells. These pathways included negative regulation of the inflammatory response and the oxidative stress induced intrinsic apoptotic signaling pathway. The DNA repair and chromosome organization pathways were also down-regulated in RS cells (Fig. S4B), which is in consistent with previous reports (Campisi, 2013; Chandra et al., 2015).

In summary, we demonstrated that unlike quiescent cells and ionizing radiation induced senescent cells, replicatively senescent cells are more heterogeneous. Some replicatively senescent cells produce and secrete more SASP which can induce paracrine senescence, leading to tumorigenesis, angiogenesis and metastasis. Identifying those cell-specific type markers with different approaches, including both experimental and bioinformatic, would greatly advance our understanding of senescence biology and lay the foundation of precisely eliminating senescent cells. In contrast to replicatively senescent cells, SIPS cells are more homogeneous. We propose that the relatively high homogeneity of parental young cells at PD38 and the type of stress inducing SIPS are major reasons causing the low heterogeneity in SIPS. However, due to the complex contexts *in vivo*, whether SIPS cells *in vivo* are heterogeneous remains to be further determined.

## Electronic supplementary material

Below is the link to the electronic supplementary material.

Supplementary material 1 (PDF 1408 kb)
