# Supplementary material for: Single senescent cell sequencing reveals heterogeneity in senescent cells induced by telomere erosion
Source: Protein Cell. 2018 Nov 12;10(5):370–5. doi: 10.1007/s13238-018-0591-y (PMC6468032; doi:10.1007/s13238-018-0591-y)
Supplement: Supplementary file 1 — Supplementary material 1 (PDF 1408 kb) [file 13238_2018_591_MOESM1_ESM.pdf]

Supplemental Material

Material and Methods

Cell culture. Human foreskin fibroblasts HCA2 were cultured in MEM (Hyclone, Cat. #SH30234) supplemented with 10% fetal bovine serum (Life Technologies, Cat. #16000), 1% nonessential amino acid (Hyclone, Cat. #SH3023801) and 1% penicillin/streptomycin (Hyclone, cat. #SV30010) at 37 ℃ with 3% O_2 and 5% CO_2. The cells were counted on a Millipore Muse machine (Hayward, CA, USA) and the PD number was calculated as previously reported[1,2].

X-ray irradiation. Cells (5×10^5) were seeded onto 10cm dish, grown for two weeks to induce quiescence. Then the quiescent cells were treated with the dose of X-ray at 50 Gy. Cells were cultured in the incubators for twenty days before Drop-seq, β-gal staining and EdU incorporation assay.

β-gal staining. Cells were fixed in fixation buffer (2% formaldehyde, 0.2% glutaraldehyde in PBS) for 5 min at room temperature. After washing twice with PBS, cells were stained in staining buffer (1 mg/ml X-gal in dimethylformamide, 40 mM citric acid/Na phosphate buffer, 5 mM potassium ferrocyanide, 5 mM potassium ferricyanide, 150 mM sodium chloride, 2 mM magnesium chloride) overnight at 37 ℃. Then followed by imaging on a Zeiss inverted microscope (Goettingen, Germany).

EdU incorporation assay. Cells were seeded onto 6-well plates at a density of 1×10^5. After 24 hours, cells were incubated with 10 μM EdU. Two hours later, cells were harvested for FACS analysis using a Click-iT EdU Assay Kit (Invitrogen, Waltham, MA, USA, C10634).

Drop-seq Library preparation. Cells were washed with 1×PBS + 0.01% BSA, filtered through a 40-μm cell strainer (Corning, Cat #352340) and resuspended in 1×PBS + 0.01% BSA + 1 U/μl RNAse inhibitor (Takara, Cat #2313A) to a concentration of 100,000 cells/ml. Barcoded drop-seq beads (Barcoded Beads SeqB[3]; Chemgenes) were washed with Drop-seq lysis buffer (DLB[3]; 6% Ficoll PM-400, 0.2% Sarkosyl, 20 mM EDTA (Life Technologies), 200 mM Tris-HCl, pH 7.5 and 50 mM DTT (where DTT is added fresh)) and resuspended in DLB at a concentration of 120,000 beads/ml. The cells and barcoded beads were loaded into 2.5ml syringes, connected to microfluidic PDMS devices (~120 μm) and flown at 3 ml/h with oil (Biorad, Cat #186-4006) at 14ml/h, using

a self-built Drop-seq setup according to Macosko et al. 2015 (Online-Dropseq-Protocol-v.-3.1, http://mccarrolllab.com/dropseq/). Droplets of about 1nl in size were generated and collected. After collection, droplets were broken using Perfluoro-1-octanol (Klamar) and beads with captured RNA were reverse transcribed and exonuclease-treated as described[3]. cDNA libraries were amplified by apportioning 2000 beads (~100 STAMPs) into each PCR tube (50 µl volume; 4 + 14 cycles), purified with 0.6×volumes of AMPure XP beads (Beckman Coulter, A63881) and quantified by Qubit dsDNA HS Assay kits. 5 ng of cDNA of each tube were pooled together. To prepare 3'-end cDNA libraries, 500pg cDNA were fragmented and amplified using Nextera XT tagmentation reactions according to the manufacturer's instructions except that 200 nM of the custom primers P5_TSO and Nextera_N7xx were used replacing the kit's provided primers[3]. The libraries were purified twice with 0.6×AMPure XP beads and once with 1×AMPure XP beads, quantified and sequenced (PE150) on Illumina HiSeq X10 sequencer using the custom primer Read1CustomSeqB.

Drop-seq Data Processing and Analysis. Raw reads which barcode base quality were less than 10 would be filtered. After removal of 5′ end TSO adaptor sequence and 3′ end poly(A) tails, reads were aligned to human (GRCh37) genome by STAR v2.5.3a with default parameter. Duplicated reads were grouped by UMI. Then we got the expression matrix. Cell with more than 500 detected genes and mitochondrial percentage less than 10% were used in downstream analysis. We used Seurat software[4] to perform cluster and dimension reduction. In order to accelerate the downstream computational analysis. We only perform scale on highly variable genes by number of genes, percentage of mitochondrial gene expression and batch. Significant principal components were determined by jackStraw procedure. We also used another method which draw a plot of the standard deviations of the principle components and find a threshold where there is a clear elbow in the plot. Then we identify significant principal components. Finally, we run non-linear dimensional reduction (tSNE) on statistically significant principal components. Differentially expressed genes analysis were based on Wilcoxon rank sum test in Seurat software.

Gene set enrichment analysis (GSEA) [5] was performed on a pre-ranked list based on adjust P-value and detect enriched pathways in Gene ontology biological process, Kyoto

Encyclopedia of Genes and Genomes (KEGG) and Reactome Pathway Database (REACTOME) gene sets which downloaded from Molecular Signature Database (MSigDB). Pathways which nom p-val < 0.05 were selected as significantly enriched pathway.

Data availability. The single cell mRNA sequencing data had been deposited to the NCBI Gene Expression Omnibus (GEO) database which can be accessed by accession number GSE119807.

Reference

1. Seluanov, A., Mittelman, D., Pereira-Smith, O. M., Wilson, J. H. & Gorbunova, V. DNA end joining becomes less efficient and more error-prone during cellular senescence. *Proc. Natl. Acad. Sci. U.S.A.* **101,** 7624–7629 (2004).
2. Mao, Z. *et al.* Sirtuin 6 (SIRT6) rescues the decline of homologous recombination repair during replicative senescence. *Proc. Natl. Acad. Sci. U.S.A.* **109,** 11800–11805 (2012).
3. Macosko, E. Z. *et al.* Highly Parallel Genome-wide Expression Profiling of Individual Cells Using Nanoliter Droplets. *Cell* **161,** 1202–1214 (2015).
4. Carrano, A. C., Mulas, F., Zeng, C. & Sander, M. Interrogating islets in health and disease with single-cell technologies. *Molecular Metabolism* **6,** 991–1001 (2017).
5. Subramanian, A. *et al.* Gene set enrichment analysis: A knowledge-based approach for interpreting genome-wide expression profiles. *Proceedings of the National Academy of Sciences* **102,** 15545–15550 (2005).

Supplementary Figure 1. Senescence were confirmed by β-gal staining and EdU incorporation assay.

A. Representative pictures and statistical results of β-gal staining on the cells of PD48 control cells and replicatively senescent cells. Analysis of cell division rates on the cells of PD48 control cells and replicatively senescent cells using the EdU incorporation assay.

B. Representative pictures and statistical result of β-gal staining on the cells of PD38 control cells and 50 Gy X-ray induced senescent cells of PD38 control cells. Analysis of cell division rates on the cells of PD38 control cells and 50 Gy X-ray induced senescent cells of PD38 control cells using the EdU incorporation assay.

Supplementary Figure 2. Droplet-based scRNA-seq reveals some marker

genes.

A. Heatmap showing expression of the top ten marker genes for each cluster. Gene expression were shown as Z-score from purple (low expression) to yellow (high expression).

B. Cell cycle associated genes MKI67, TOP2A and CENPE's expression of each cluster.

Supplementary Figure 3. Transcriptome change of HCA2 fibroblasts with replicative senescence.

A. Number of significantly differentially expressed genes of each cluster.

B. Venn diagram showing numbers of up- and down-regulated genes for each cluster.

C. Number of significantly enriched pathways of each cluster.

D. Enriched pathways of down-regulated genes of cluster1-3. Left table: Cluster3-specific enriched pathways of cluster3' down-regulated genes. Bottom table: Cluster2 and cluster3 common enriched pathways of cluster2's and cluster3's down-regulated genes. Right table: Cluster2-specific enriched pathway of cluster2's down-regulated genes. Upper right table: Cluster1-specific enriched pathway of cluster1's down-regulated genes.

Supplementary Figure 4. Transcriptome difference between telomere erosion and ionizing radiation induced senescence

A. Enriched pathways of up-regulated genes of telomere erosion and ionizing radiation induced senescence. Bottom table: Telomere erosion induced senescence-specific enriched pathways of its up-regulated genes. Right table: Ionizing radiation induced senescence-specific enriched pathways of its up-regulated genes.

B. Enriched pathways of down-regulated genes of telomere erosion and ionizing radiation induced senescence. Bottom table: Telomere erosion induced senescence-specific enriched pathways of its down-regulated genes. Right table: Ionizing radiation and telomere erosion induced senescence common enriched

pathways of their down-regulated genes.

Supplementary Figure 1

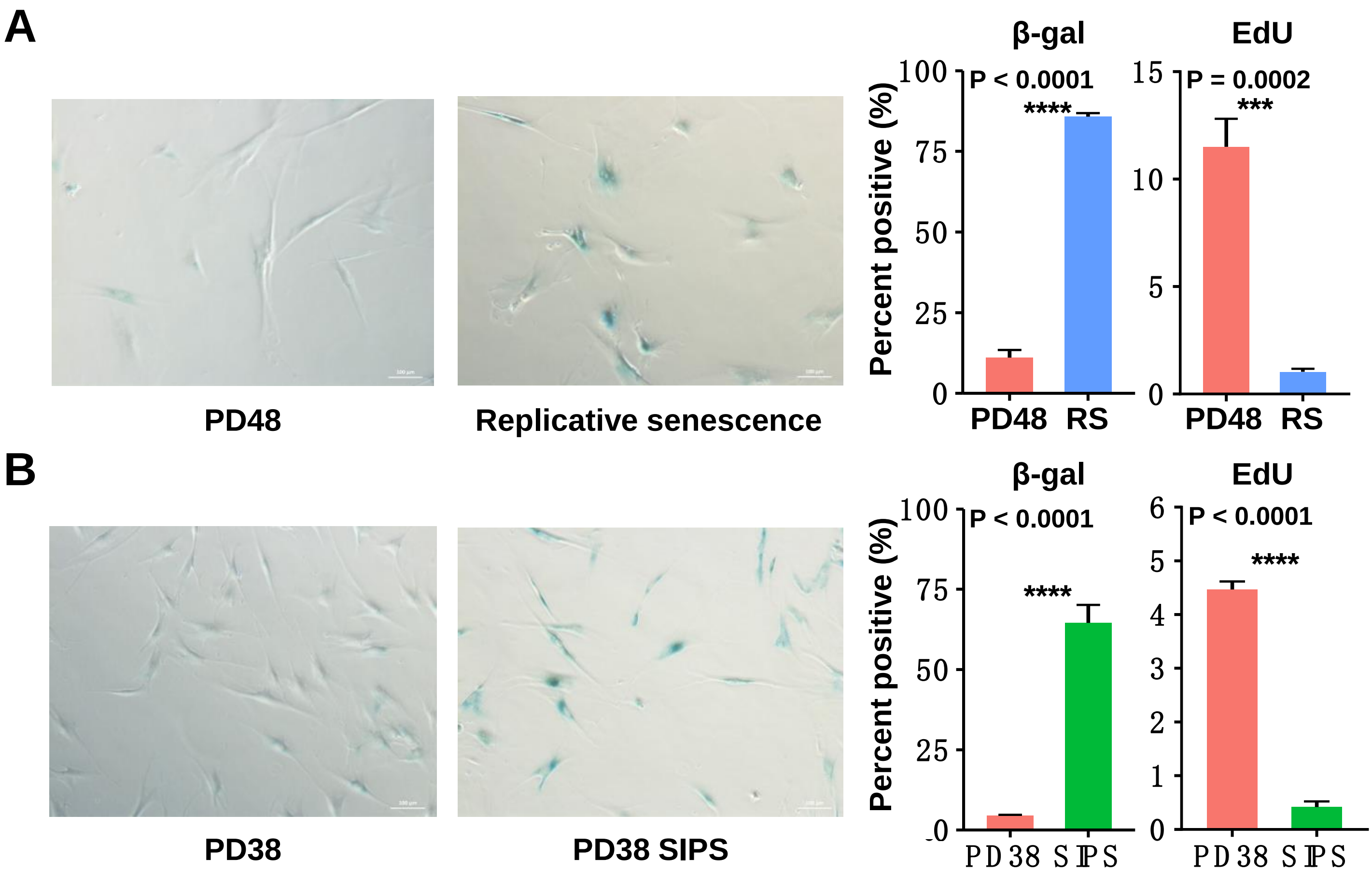

Supplementary Figure 2

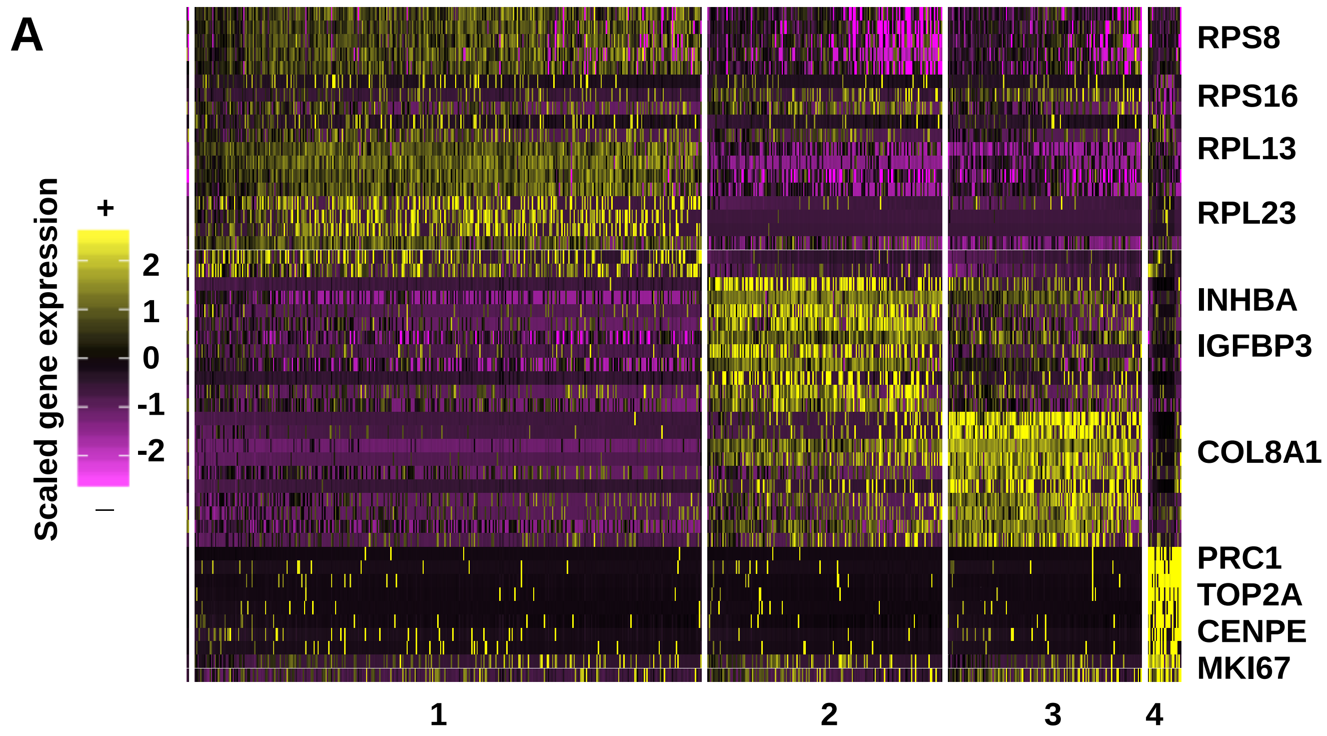

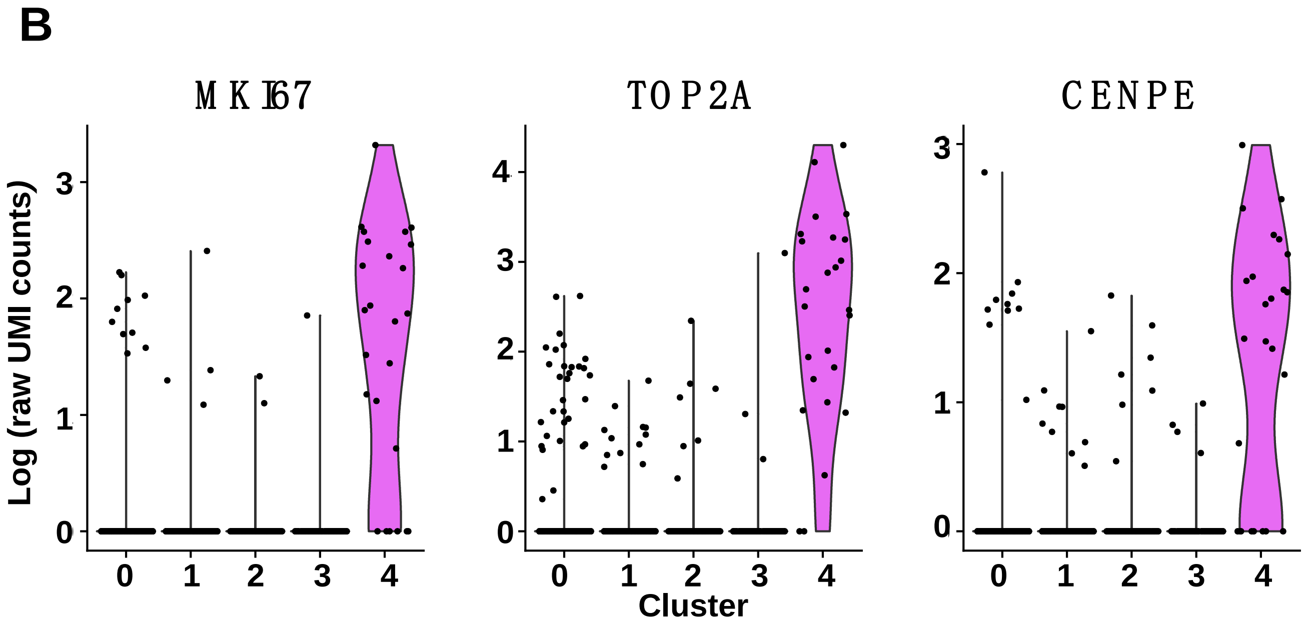

Supplementary Figure 3

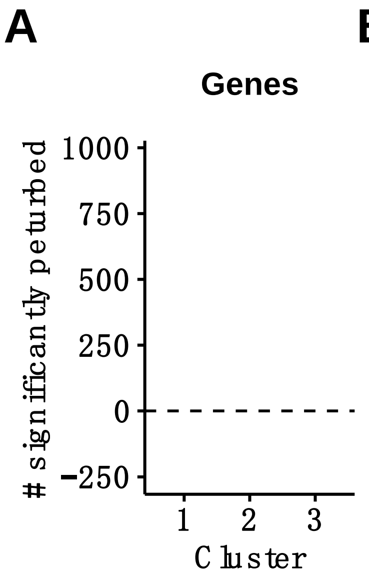

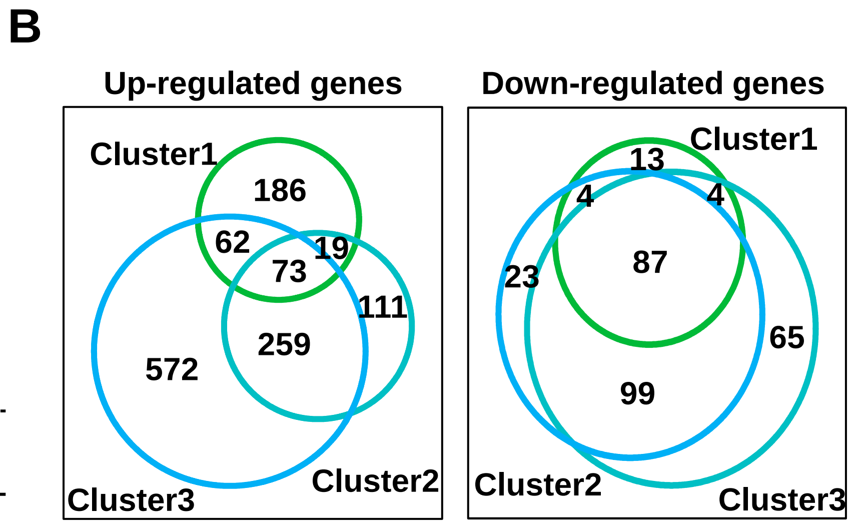

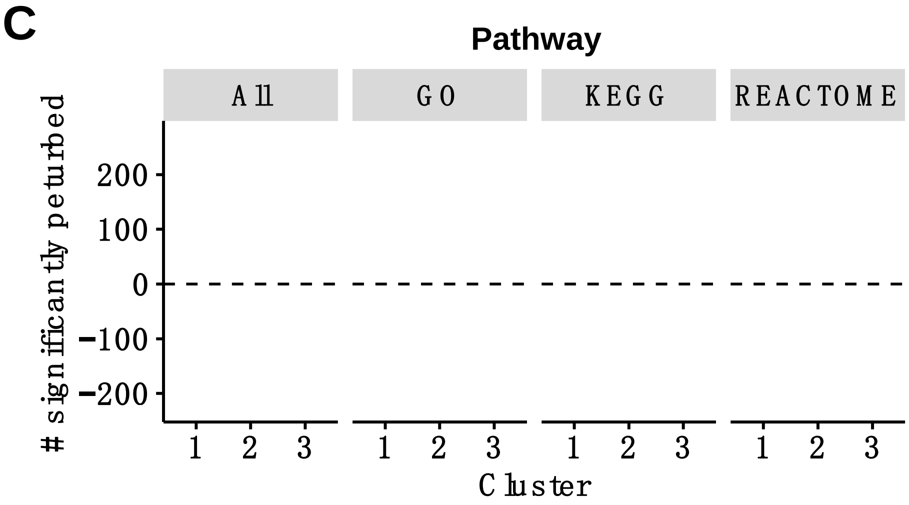

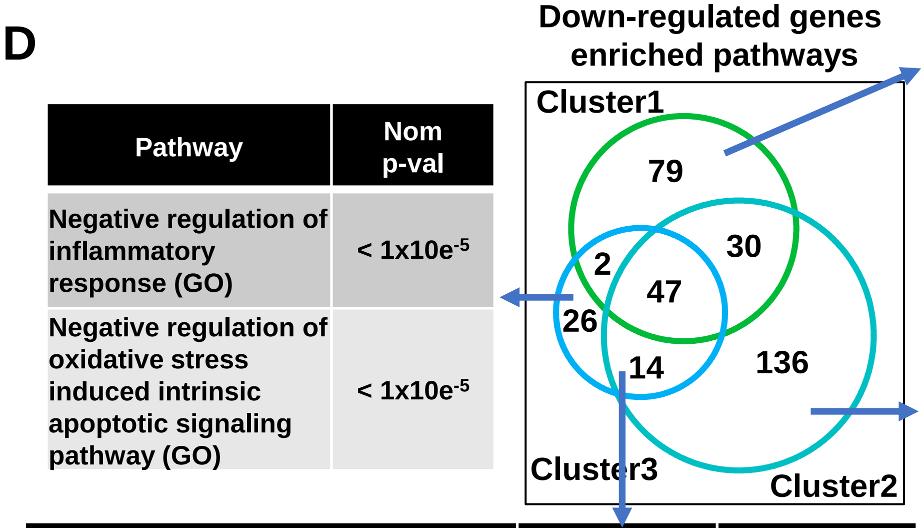

Pathway	Nom p-val
Lysine degradation (KEGG)	< 1x10e-5
Base excision repair (KEGG)	< 1x10e-5
RNA methylation (GO)	< 1x10e-5
Telomere maintenance via telomerase (GO)	< 1x10e-5

Pathway	Nom p-val
Negative regulation of inflammatory response (GO)	< 1x10e-5
Negative regulation of oxidative stress induced intrinsic apoptotic signaling pathway (GO)	< 1x10e-5

Pathway	Nom p-val
Regulation of autophagy (KEGG)	< 1x10e-5
Innate immune response activating cell surface receptor signaling pathway (GO)	8x10e-3
Activation of NF-κB in b cells (REACTOME)	1.7x10e-2
Regulation of apoptosis (REACTOME)	1.7x10e-2
Positive regulation of chromosome organization (GO)	2.7x10e-2
Dna repair (GO)	3.2x10e-2

Pathway	Nom p-val (Cluster2)	Nom p-val (Cluster3)
Ribosome biogenesis (GO)	< 1x10e-5	< 1x10e-5
Translation (REACTOME)	< 1x10e-5	< 1x10e-5
Metabolism of protein (REACTOME)	< 1x10e-5	< 1x10e-5
Ribosome (KEGG)	1.2x10e-04	< 1x10e-5

Supplementary Figure 4

A

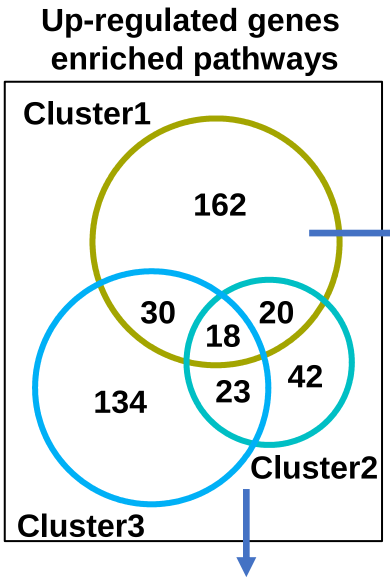

Pathway	Nom p-val
Regulation of protein catabolic process (GO)	< 1x10e^{-5}
Negative regulation of molecular function (GO)	< 1x10e^{-5}
Negative regulation of catalytic activity (GO)	< 1x10e^{-5}
DNA integrity checkpoint (GO)	1.6x10e^{-3}
Signaling by NOTCH1 (REACTOME)	1.3x10e^{-2}
VEGF signaling pathway (KEGG)	2.3x10e^{-2}

Pathway	Nom p-val
Positive regulation of cytokine production (GO)	5.9x10e^{-3}
IL1 signaling (REACTOME)	9.1x10e^{-3}
Angiogenesis (GO)	1.2x10e^{-2}
Positive regulation of TNF superfamily cytokine production (GO)	1.3x10e^{-2}

B

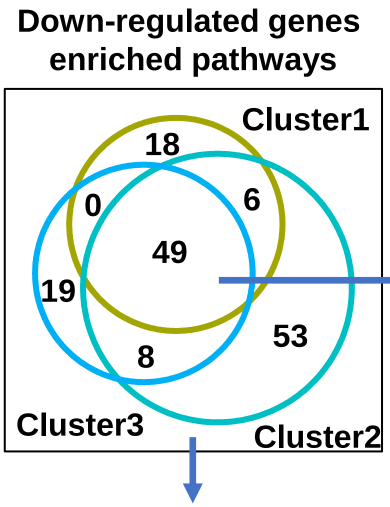

Pathway	Nom p-val (Ionizing radiation)	Nom p-val (telomere erosion)
Ribosome biogenesis (GO)	< 1x10e^{-5}	< 1x10e^{-5}
Translation (REACTOME)	< 1x10e^{-5}	< 1x10e^{-5}
Ribosome (KEGG)	< 1x10e^{-5}	< 1x10e^{-5}
Metabolism of protein (REACTOME)	< 1x10e^{-5}	< 1x10e^{-5}

Pathway	Nom p-val
Negative regulation of inflammatory response (GO)	< 1x10e-5
Negative regulation of oxidative stress induced intrinsic apoptotic signaling pathway (GO)	< 1x10e-5
DNA repair (REACTOME)	2.9x10e-02
Chromosome organization (GO)	3.7x10e-02
